# Supplementary material for: A high-resolution mRNA expression time course of embryonic development in zebrafish
Source: eLife. 2017 Nov 16;6:e30860. doi: 10.7554/eLife.30860 (PMC5690287; doi:10.7554/eLife.30860)
Supplement: Supplementary file 6. [file elife-30860-supp6.zip › biolayout-clusters-files/Cluster174.html]

Cluster174


# Cluster174: Detail

### Go to ZFA detail

## GO

| GO ID | Description | Domain | Annotated | Expected | Observed | Adjusted p-value | Genes | Ensembl IDs |
| --- | --- | --- | --- | --- | --- | --- | --- | --- |
| GO:0006400 | tRNA modification | biological\_process | 27 | 0.02 | 2 | 0.036 | trmt1 adat2 | ENSDARG00000010978 ENSDARG00000041944 |
| GO:0030686 | 90S preribosome | cellular\_component | 20 | 0.01 | 2 | 0.020 | pwp2h srfbp1 | ENSDARG00000037109 ENSDARG00000075323 |
